# Improving the greenness of enterprise supply chains by designing government subsidy mechanisms: based on prospect theory and evolutionary games

**DOI:** 10.3389/fpsyg.2023.1283794

**Published:** 2023-10-23

**Authors:** Li Hou, Yiming Zhang, Chunlin Wu, Jinbo Song

**Affiliations:** ^1^School of Economics and Management, Ningbo University of Technology, Ningbo, China; ^2^School of Economics and Management, Dalian University of Technology, Dalian, China; ^3^School of Economics and Management, Beihang University, Beijing, China; ^4^Beijing Key Laboratory of Emergency Support Simulation Technologies for City Operations, Beihang University, Beijing, China

**Keywords:** green supply chain, government subsidies, prospect theory, evolutionary game, reward and punishment mechanisms

## Abstract

Fostering sustainable development through green supply chains is of paramount significance. Government subsidies emerge as a successful strategy for motivating businesses to actively participate in such eco-friendly practices. This study employs prospect theory and an evolutionary game model to analyze the transition toward carbon peaking and neutrality while promoting the expansion of highly sustainable businesses. By exploring the decision-making processes of businesses and governments regarding sustainability, we develop an evolutionary game-based decision model to assess the impact of government subsidies on businesses engaged in green supply chains. Through numerical simulation obtained via MATLAB, we examine various factors influencing the evolution of the game system between green supply chain businesses and the government. Additionally, we investigate how government incentives impact the decision-making behavior of green supply chain businesses. Our findings indicate that governmental fines can effectively encourage the adoption of green supply chains. Furthermore, moderate government subsidies incentivize enterprises to opt for sustainable supply chains, benefiting both the government and businesses. However, providing hefty government subsidies not only fails to encourage the adoption of green supply chains but also incurs costs for the government, without yielding any positive change in the businesses’ approach. By incorporating evolutionary game theory and prospect theory, this study contributes to the body of knowledge on government-supported green supply chains, offering incentive programs tailored to the real-world conditions faced by businesses while demonstrating practical application values.

## 1. Introduction

As global climate change intensifies, most countries have realized the need to shift from traditional economic growth models to green development paths. Carbon dioxide emissions are the leading cause of global warming. More than 20% of the world’s greenhouse gas (GHG) emissions come from the 2,500 largest multinational corporations’ supply chains (World Bank, 2022). Numerous organizations have incorporated environmental factors into the project governance of their supply chains as a result of increased awareness, visibility, and stakeholder pressure (Song J. et al., 2022a). Supply chain corporations have played a significant role in these endeavors by executing investment decisions that are less detrimental to the environment than their existing equivalents (Tian et al., 2022). Such a strategy effectively mitigates environmental impacts throughout the entire supply chain process. And it is possible lowering environmental stress via sustainable supply chains. Therefore, green supply chains hold the potential to significantly alleviate or even reverse the environmental impact caused by human activities. In the face of intense international market competition, growing uncertainty in economic demand, and continuous technological innovation, green supply chains have become essential for supply chain enterprises.

Government subsidy mechanisms play a critical role in fostering enterprise involvement in green supply chains (Wang and Zhang, 2023). Stakeholders within green supply chain enterprises exhibit bounded rationality, and such enterprises adapt their strategies in response to government subsidy actions, which is more consistent with the actual behavioral characteristics of the players (Zhu et al., 2022). Therefore, the government can facilitate the development of a green economy, industrial transformation, and green supply chains through policy guidance and financial support. Currently, due to the defects in market mechanisms and constraints of individual business interests, the development of green supply chains faces numerous obstacles. For instance, small rural retailers in developing countries face challenges related to supply chain reform and technology management (Guo et al., 2022b). In this context, decision-making for green supply chain enterprises involves various complexities, such as cost reduction, interlinking interests, and adapting to dynamic market environments.

Considering the increasing complexities in logistics and supply chains in the current global business climate, understanding decision-making intricacies becomes imperative. Consequently, adopting more suitable methods for making green supply chain decisions has become increasingly vital. In the green supply chain, the decision-making behavior of government management and enterprise operation is often affected by subjective judgment and value perception (Hu et al., 2023). Prospect theory, recognized as a well-established framework for depicting risky decision-making, emerges as a mature decision theory (Tversky and Kahneman, 1992). This theory deviates from expected utility theory in two crucial aspects. First, utility under Prospect theory is assigned to gains and losses considering a reference point and not the final acquisition. This feature is known as reference dependence. Second, losses “loom larger” than gains of the same size, and this phenomenon is referred to as loss aversion. Prospect theory argues that decision-makers value gains and losses distinctively, with emphasis on perceived gains than on perceived losses (Aydogan, 2021). This theory acknowledges the crucial role of emotional states and rational capabilities within the intricate framework of green supply chain decision-making, thereby equipping enterprise decision-makers to make more astute choices (Heutel, 2019). Prospect theory emerges as an essential tool in promoting and guiding business and government entities through complex decision-making scenarios (Cai et al., 2023). By considering the decision-making processes of both sectors, it provides profound insights into diverse decision environments and significantly promotes the advancement of green practices in corporations.

Furthermore, integrating prospect theory with evolutionary games can optimize its application and provide a more comprehensive representation of the risky decision process. Some scholars have integrated evolutionary game and prospect theory to study human behavior in diverse fields (Golrezaei et al., 2021). Evolutionary games, an essential aspect of game theory, facilitate the comprehension of complex systems and cooperative behavior evolution (Killingback and Doebeli, 1996). This aspect renders them highly potent in modeling real-world economic issues and supporting decision-making processes undertaken by economists and policymakers (Schwerter, 2023). The versatility of evolutionary games extends to various domains, such as economics, management, political science, and social and biological evolution, addressing problems such as pricing wars between businesses, government-business cooperation, market volatility, and competition (He and Strub, 2022). The prospect theory was integrated into an evolutionary game framework to scrutinize the decision-making processes of businesses and governments within the realm of a green supply chain. The objective of this study, which combines evolutionary game theory and prospect theory, revolves around examining the decision-making process concerning government subsidies and green supply chains. It seeks to empower businesses and governments in enhancing their sustainability efforts within the context of green supply chains. This study analyzes government policies in diverse scenarios and their impact on business decisions, presenting the perspectives of both the government and businesses. By leveraging prospect theory and evolutionary gaming, effective decision-making support can be provided to meet market demands amid the increasingly complex supply chain environment.

This paper employs evolutionary game theory and mathematical modeling to explore the effects of two decisions (subsidization and penalties) based on their choice of green supply chains. The goal is to better address the challenges faced by green supply chain enterprises and the government in their decision-making processes. We explore the effect of irrational psychology on decision-making entities within green supply chain enterprises operating in collaboration with the government. This study contributes in three ways by integrating prospect theory, evolutionary game, and a review of the existing literature. First, our study broadens the theoretical understanding of green supply chains by introducing the perspective of psychological and behavioral factors. Second, our research has improved the design of government subsidy mechanisms by advancing the field of game theory research in the evolution of green supply chain subsidies. Third, we demonstrate the interactive relationship between green supply chain decisions and governmental subsidies and identify the specific factors of green supply chain practices that are favourable to sustainability. Our research contributes to the formulation of effective government subsidy strategies by aligning them with the varied strengths of subsidies and associated punitive measures. The central theme focuses on government’s strategies to incentivize businesses to adopt eco-friendly, green supply chains.

## 2. Literature review

### 2.1. Prospect theory

Prospect theory, a descriptive decision model rooted in psychology and behavior, finds application in various domains such as economics, political science, and psychology. It serves as a guiding framework for decision-making involving risks, ranging from personal choices to financial matters and policy welfare (Ruggeri et al., 2020). The influence of prospect theory on political science and decision-making in international relations has been subject to investigation. Its usefulness in political science has been confirmed, and further in-depth studies are being pursued. Additionally, researchers have analyzed the relative significance of prospect theory components in economic decision-making by aggregating research over four decades (Cheng and Cheng, 2023). Outside the laboratory, prospect theory finds relevance in settings where attitudes toward risk play a pivotal role. Consequently, an increasing number of scholars are applying prospect theory as a psychological and behavior-based descriptive model for research in various economic fields, with implications for decisions involving risk (Uppari and Hasija, 2019). Furthermore, prospect theory plays a vital role in diverse contexts, including agriculture and business negotiations. For instance, research has shown a significant connection between farmers’ risk preferences, risk perceptions, and observed risk behavior in the context of climate change. Farmers who align with the prospect theory hypothesis are more likely to perceive higher risks associated with climate change (Villacis et al., 2021). In the realm of commercial negotiations, the impact of loss aversion on renegotiation has been studied through mathematical models based on prospect theory. These studies reveal that loss aversion leads to sticky and inefficient renegotiation outcomes (Trivella et al., 2021).

Moreover, prospect theory is employed in logistics decision-making, particularly in emergency logistics. The SF-GRA multi-attribute group decision-making method, based on cumulative effect theory, has been applied to the selection of emergency material suppliers (Katok and Villa, 2022). This approach evaluates supplier scores on multiple attributes using the SF-GRA method, converts them into utility values through cumulative effect theory, and then ranks the suppliers using the TOPSIS method to select the optimal supplier (Zhang et al., 2022).

### 2.2. Evolutionary game

The evolutionary game is a mathematical model that elucidates the evolution of individual behavior in society (Jung and Kouvelis, 2022). It is built upon the principles of Mendelian genetics and draws upon the evolutionary theory in ecology, enabling the study of strategy selection regularity and the evolution of group behavior among participants in the game process (Long et al., 2021). At the heart of the evolutionary game lies the concept of an “evolutionary stable strategy.” This strategy, after several rounds of the game, becomes widely adopted and cannot be easily replaced by alternative strategies, making it evolutionarily stable. The theory of evolutionary games describes the interaction of individuals within a group, asserting that individual behavior is influenced by the behavior of others in the group, and follows certain evolutionary laws (Kwon, 2022). In this context, individuals may exhibit cooperative or non-cooperative behaviors, with different consequences for individual and group payoffs (Li X. et al., 2020). As an essential interdisciplinary research field, evolutionary game theory has garnered significant attention from scholars. It holds substantial importance for comprehending the evolutionary mechanisms of complex systems and cooperative behavior. Furthermore, evolutionary game theory has been applied to investigate dynamic reward and punishment schemes in green building development incentives, revealing that in competitive construction markets, such schemes can encourage the adoption of more environmentally friendly and sustainable building designs, enhancing their market competitiveness (Meng et al., 2021b).

Intra-enterprise collusion has also been studied through the lens of the tripartite evolutionary game of railroad safety regulation (Birge et al., 2023). These studies show that intra-enterprise collusion can erode trust between regulators and railroad enterprises, thereby affecting the effectiveness of railroad safety regulation (Tang et al., 2021). Additionally, researchers have reviewed the application of game theory in green supply chain management (Agi et al., 2021). Long et al. (2021) utilized evolutionary game theory to analyze the behavior of green-sensitive parties in green supply chains, considering interactions among green suppliers, green buyers, and non-green buyers.

### 2.3. Green supply chain

A supply chain is a network structure established by a core enterprise, encompassing related manufacturing, assembly, distribution, and retail enterprises interconnected through information flow, material flow, and capital flow. Through this network, enterprises transform raw materials into products, which are then sold to end-users (Song S. et al., 2022). A green supply chain integrates the concepts of green manufacturing, product life cycle, and extended producer responsibility into the business process of enterprises based on the traditional supply chain (Stumpf et al., 2023). This integration aligns the economic benefits of enterprises with resource conservation, environmental protection, and human health and safety requirements (Birge et al., 2023). Implementing green supply chain management is an effective strategy for enhancing the competitiveness of enterprises and achieving green and sustainable development (Zhou et al., 2021).

Green supply chain management is a management approach emphasizing environmental and social responsibility, which has garnered significant attention from researchers over the past 30 years (Nematollahi and Tajbakhsh, 2020; Roemer et al., 2023). A review of the literature on decision theory in sustainable supply chain management suggests that enterprises are increasingly adopting prospect theory in green supply chain decision-making to enhance its effectiveness (Zhu et al., 2022). Green supply chains incorporate environmental considerations into various supply chain practices, including product design and development, procurement, manufacturing, logistics, and end-of-life management (Li G. et al., 2020). The ultimate goal of green supply chains is to minimize environmental impact while simultaneously reducing costs, improving efficiency, and enhancing customer satisfaction (Song et al., 2023).

Recent research on green supply chains has focused on the trade-offs between different cost factors and environmental impacts in green supply chains to design greener and sustainable supply chain networks. Some researchers delved into the incentives and shared responsibility for emissions in supply chains, arguing that considering emission responsibility is crucial for achieving global environmental goals. Adopting incentive mechanisms can motivate supply chain participants to reduce emissions (Gopalakrishnan et al., 2021). To promote green product process innovation, an approach based on closed-loop supply chains and remanufacturing has been proposed by some researchers. This approach optimizes product design, production, and remanufacturing processes to maximize resource utilization and environmental protection while improving the enterprise’s economic efficiency (Park et al., 2022). Scholars also discuss key challenges and responses related to supply chain collaboration, technological innovation, and policy support. Future research directions include an in-depth study of supply chain synergy effects and remanufacturing process optimization to further sustainable development goals (Chai et al., 2021). Researcher integrated green supply chain operations and hospital environmental performance were examined in relation to the effects of big data analytics and artificial intelligence. According to the study, big data analytics and artificial intelligence applications may considerably boost the effectiveness and quality of green supply chains while also assisting in the reduction of resource waste and environmental degradation (Benzidia et al., 2021).

### 2.4. Government subsidies for green supply chains

Researchers are increasingly focusing on the role and impact of government subsidies in green supply chains. Some have examined the important interaction between subsidies and regulations in the market and presented key findings. The effectiveness of these measures depends on market circumstances (Wang et al., 2021). Subsidies and regulations are essential to correct market failures, but caution is needed when competition exists to avoid negative impacts on it (Zhang et al., 2023). Thus, the government must carefully consider the interplay between subsidies and regulations and select appropriate policies based on market conditions to maximize societal benefits (Wang and Zhang, 2023). From the perspective of carbon taxes, studies have explored the environmental and economic performance of closed-loop supply chains. Others have investigated the impact of the emissions intensity of new and remanufactured products on the overall emissions (Dou and Cao, 2020). These results highlight the importance of government involvement and promotion of green practices in organizations, as well as the significant role of environmental awareness in society for building a strong green supply chain management framework.

Scholars have provided effective green supply chain management strategies for enterprises and policymakers (Lo et al., 2022). They have discussed green subsidy models and pricing strategies in capital-limited supply chains. The concept and types of green subsidies are introduced, followed by an exploration of their impact on various supply chain participants (Gopalakrishnan et al., 2021). Additionally, the significance of pricing strategies for green supply chains is discussed, including the influencing factors and decision-making process (Huang et al., 2020). Studies have also examined the impact of government involvement, enterprise green investment, and consumer green preferences on supply chain coordination for the oil supply chain. Research has demonstrated that government encouragement of enterprise green investment and consumer advocacy of green products contributes to improved supply chain coordination (Zhang and Yousaf, 2020). Regarding taxation, scholars have explored environmental governance strategies for supply chains in the context of tax and subsidy interactions and found that flexible tax and subsidy strategies should be adopted by governments to address the varying needs of different enterprises and consumers, ultimately achieving sustainable development goals (Li Y. et al., 2020).

Researchers have also explored the incorporation of government subsidies into optimization models for redesigning optimal trauma care networks (Liu et al., 2023). Additionally, they have used evolutionary game models of green innovation stakeholders and simulation methods to analyze them. The findings emphasize the significant impact of stakeholder behavior and decisions on sustainable development in the field of green innovation (Guo et al., 2022a). To promote sustainable development, positive actions should encourage stakeholders to adopt cooperative strategies (Aydinliyim et al., 2022). Government intervention and regulation play a crucial role in fostering green innovation and sustainable development (Zhou et al., 2022).

## 3. Construction of the evolutionary game model

**Figure 1:**
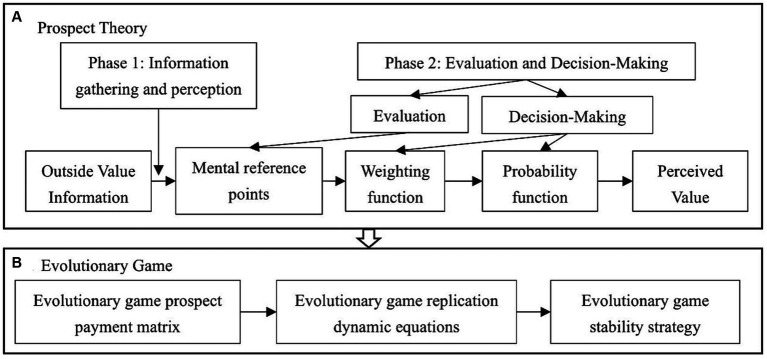
Game analysis process of government and green supply chain enterprises.

In this study, we combine evolutionary game theory with prospect theory and expand upon the evolutionary game model of governmental and green supply chain businesses. First, we applied prospect theory to analyze the perceived prospect value of enterprises and governments under different decisions. The model analyzes and calculates the perceived prospect value of each strategy for the government and enterprises, considering the two stages of prospect theory. Subsequently, we conducted an evolutionary game analysis based on the prospect value for each decision subject. Figure 1 illustrates the analysis process of the model in this paper.

### 3.1. Description of evolutionary game problem

The government subsidizing the green supply chain involves two key decision-makers: the government itself and the enterprises. These two players can be viewed as participants in a game, wherein neither acts rationally. Initially, each business must determine whether to adopt a “high green” or “low green” approach, with the chosen strategy significantly impacting the costs and benefits of the firm. Simultaneously, the government faces the decision of either imposing restrictions or offering incentives to influence behavior. The strategic decisions taken by the government significantly influence not only its own cost–benefit dynamics but also the choices pursued by businesses. The interaction between these strategic decisions from both the government and the enterprise creates a game process.

For the government and the enterprise to achieve a desirable outcome, they must strive to reach a Nash equilibrium point. In this equilibrium, neither party can unilaterally modify its plan to gain greater rewards, leading to a balanced and mutually beneficial arrangement. The model depicts the game between the enterprises and the government as a repeated game. This means that they engage in multiple rounds of decision-making. In each round, they base their new decisions on the results of the previous round. As the number of rounds increases, the strategies employed by both parties gradually stabilize, eventually leading to a dynamic equilibrium state.

### 3.2. Basic model assumptions

#### 3.2.1. Assumption 1

There are two decision makers, namely enterprise *x* and government *y*, both of whom are not fully rational when considering whether to subsidize green supply chains. Within the green supply chain, there are two types of enterprise strategies: the “high green” strategy and the “low green” strategy. The government has two policy options: the incentive policy and the regulatory policy. The incentive policy involves the government providing incentives and subsidies to environmentally friendly enterprises, while the regulatory policy entails the government enforcing environmental protection supervision and imposing penalties on non-compliant enterprises. Initially, the proportions of the two strategies are represented by *p* and 1−*p*, while the proportions of the two policy options are represented by *q* and 1−*q*. When an enterprise adopts the “high green” strategy, it incurs a benefit of *R*_1_ and a cost of *C*_1_. Conversely, when an enterprise adopts the “low green” strategy, it incurs a benefit of *R*_2_ and a cost of *C*_2_. When the government adopts the incentive policy, it offers a subsidy of *G* to high green enterprises. In contrast, when the government adopts the regulatory policy, it imposes a penalty of P on low green enterprises, it experiences a benefit of *G*_1_ and a cost of *K*_1_ for the incentive policy, and a benefit of *G*_2_ and a cost of *K*_2_ for the regulatory policy. The prospective value of environmental benefits to the government after enterprises implement the “high green” strategy is represented by *U*, listed in Table 1.

**Table 1:** Variables and explanation (Assumption 1).

Symbols	Variable description	Symbols	Variable description
*R*_1_	The benefits for enterprise when they adopt a “high green” strategy	*G*_1_	The benefits to the government when it adopts incentive policy
*C*_1_	The costs for enterprise when they adopt a “high green” strategy	*K*_1_	The cost to the government when it adopts incentive policy
*R*_2_	The benefits for enterprise when they adopt a “low green” strategy	*G*_2_	The benefits to the government when it adopts regulatory policies
*C*_2_	The costs for enterprise when they adopt a “low green” strategy	K_2_	The cost to the government when it adopts regulatory policies
*G*	Actual amount of government subsidies to high green businesses when using incentives	*P*	The actual fines levied on low green businesses when the government implements regulatory policies
*U*	The prospective value of environmental benefits to the government when enterprise implement “high green” strategies		

#### 3.2.2. Assumption 2

The strategy sets of enterprise *x* and government *y* are $S_{e}$ { $s_{e}^{g}$ , $s_{e}^{n}$ } and $S_{g}$ { $s_{g}^{a}$ , $s_{g}^{b}$ }, respectively, where $s_{e}^{g}$ indicates that the green supply chain enterprise decides to impose more stringent green requirements on upstream enterprises on top of environmental compliance, i.e., the “high green” strategy, and $s_{e}^{n}$ indicates that the green supply chain enterprise decides to maintain environmental compliance, i.e., the “low green” strategy. Furthermore, $s_{g}^{a}$ indicates that the government adopts an incentive policy and $s_{g}^{b}$ indicates that the government adopts a regulatory policy. Based on the risk perception characteristics of decision makers, the prospect theory and cumulative prospect theory models can be composed as follows:


(1) $$V\left(x,p\right)=w\left(p\right)\cdot v\left(x\right)\text{,}$$

(2) $$v\left(x\right)=\{\begin{array}{c} v^{+}\left(x\right)=x^{\alpha },\;x\geq 0;\\ v^{-}\left(x\right)=-\lambda_{1}{\left(-x\right)}^{\beta },\;x<0; \end{array}\text{,}$$

and


(3) $$w\left(x\right)=\{\begin{array}{c} w^{+}\left(p\right)=\frac{p^{\lambda }}{{\left[P^{\lambda }+\left(1-P^{\lambda }\right)\right]}^{\frac{1}{\lambda }}}\;x\geq 0\\ w^{-}\left(p\right)=\frac{p^{\delta }}{{\left[P^{\delta }+\left(1-P^{\delta }\right)\right]}^{\frac{1}{\delta }}}\;x<0 \end{array}$$

where *α* and *β* is the coefficient of risk appetite, *λ_1_* is the coefficient of loss aversion, *λ* is the gain perception probability coefficient, *δ* is the loss perception probability coefficient, *p* is the actual probability of an event occurring, *w*(*p*) is the subjective weight probability, and *x* is the amount of change in the value identified by the decision maker relative to the reference point. Generally, *α* = *β* = 0.88, *λ*_1_ = 2.25, *λ* = 0.61, and *δ* = 0.69 are based on information acquired through previous experiments (Kahneman and Tversky, 1979).

Furthermore, $V_{e}$ { $\pi_{e}^{g}$ , $\pi_{e}^{n}$ } and $V_{g}$ { $\pi_{g}^{g}$ , $\pi_{g}^{n}$ } are the sets of projected revenue functions for the enterprise and government, respectively. The likelihood of having $\pi_{e}^{g}$ profit is *p_g_* for “high green” enterprises, and the probability of having zero profits is 1 – *p_g_*. The probability of having $\pi_{e}^{n}$ profit is *p_n_* for “low green” enterprises, and the probability of having zero profits is 1−*p_n_*. Accordingly, “high green” enterprises have a revenue prospect function of $\pi_{e}^{g}=V\left(s_{e}^{g},p_{g}\right)$ , while “low green” enterprises have a revenue prospect function of $\pi_{e}^{n}=\left(s_{e}^{n},p_{n}\right)$ :


(4) $$\begin{array}{c} \pi_{e}^{g}=V\left(s_{e}^{g},p_{g}\right)=w^{+}\left(p_{g}\right)v^{+}\left(R_{1}-C_{1}\right)+w^{-}\left(0\right)v^{-}\left(0\right)\\ \;=w^{+}\left(p_{g}\right)v^{+}\left(R_{1}-C_{1}\right) \end{array}$$

and


(5) $$\begin{array}{c} \pi_{e}^{n}=V\left(s_{e}^{n},p_{n}\right)=w^{+}\left(p_{n}\right)v^{+}\left(R_{2}-C_{2}\right)+w^{-}\left(0\right)v^{-}\left(0\right)\\ =w^{+}\left(p_{n}\right)v^{+}\left(R_{2}-C_{2}\right) \end{array}$$

The probability of a zero return is $1-q_{a}$ , such that the prospect function for the government implementing an incentive strategy is $\pi_{g}^{a}=V\left(s_{g}^{g},q_{a}\right)$ . The probability of a zero return is $1-q_{b}$ . Finally, $\pi_{g}^{b}=V\left(s_{g}^{n},q_{b}\right)$ represents the prospect function for the government to enact a regulatory policy:


(6) $$\begin{array}{c} \pi_{g}^{a}=V\left(s_{e}^{g},q_{a}\right)=w^{+}\left(q_{a}\right)v^{+}\left(G_{1}-K_{1}\right)+w^{-}\left(0\right)v^{-}\left(0\right)\\ =w^{+}\left(q_{a}\right)v^{+}\left(G_{1}-K_{1}\right) \end{array}$$

and


(7) $$\begin{array}{c} \pi_{g}^{b}=V\left(s_{g}^{n},q_{b}\right)=w^{+}\left(q_{b}\right)v^{+}\left(G_{2}-K_{2}\right)+w^{-}\left(0\right)v^{-}\left(0\right)\\ =w^{+}\left(q_{b}\right)v^{+}\left(G_{2}-K_{2}\right) \end{array}$$

The firm’s perceived value of the actual government subsidy, *G*, is *G*′. The firm’s perceived value of the actual government penalty, *P*, is *P*′, as follows:


(8) $$G^{\prime }=V\left(G,1\right)=w^{+}\left(1\right)v^{+}\left(G\right)+w^{-}\left(0\right)v^{-}\left(0\right)=v^{+}\left(G\right)$$

and


(9) $$P^{\prime }=V\left(P,1\right)=w^{+}\left(0\right)v^{+}\left(0\right)+w^{-}\left(1\right)v^{-}\left(P\right)=v^{-}\left(P\right)$$

The government’s perceived value of the real government subsidy, *G*, is *G*_g_, which is as follows:


(10) $$G_{g}=V\left(G,1\right)=w^{+}\left(0\right)v^{+}\left(0\right)+w^{-}\left(0\right)v^{-}\left(G\right)=v^{-}\left(G\right)$$

Table 2 lists the above variables and provides an explanation. The following presumptions were used based on this information:

1. Firm X and government Y independently select their strategies at each time point and reap the corresponding benefits.
2. The perceived benefits for both the firm and government are determined by the sum of the prospect value and the subsidy-related amount.
3. The evolution of the firm’s strategy is influenced by the strategies of neighboring enterprises and the government.
4. Similarly, the evolution of the government’s strategy is influenced by the firm’s strategy.

**Table 2:** Variables and explanation (Assumption 2).

Symbols	Variable description	Symbols	Variable description
$\pi_{e}^{g}$	Perceived value when enterprise adopt a “high green” strategy	$\pi_{g}^{a}$	Perceived value when the government adopts incentive policies
$\pi_{e}^{n}$	Perceived value when enterprise adopt a “low green” strategy	$\pi_{g}^{b}$	Perceived value when the government adopts regulatory policies
*p_g_*	The probability that a “high green” firm with return $\pi_{e}^{g}$	*q_a_*	When the government adopts an incentive policy, the probability that the government gains $\pi_{g}^{a}$
*p_n_*	The probability that a “low green” firm with return $\pi_{e}^{n}$	*q_b_*	When the government adopts a regulatory policy, the probability that the government gains $\pi_{g}^{b}$
*G*′	The perceived value of government subsidies to businesses	*P*′	The perceived value of government penalties to business and government
*G_g_*	The perceived value of government subsidies to the government		

### 3.3. Game benefit matrix

According to previous assumptions, the evolutionary game model for the enterprise and government can be expressed by the following game matrix, when the measurement strategy is { $s_{e}^{g}$ , $s_{g}^{a}$ }. According to the assumption that the enterprise selects “high green,” and the government selects the incentive policy, the perceived benefit of the enterprise is $\pi_{e}^{g}+G^{\prime }$ and the perceived benefit of the government is $\pi_{g}^{a}+U-G^{\prime }$ . When the strategy scenario is { $s_{e}^{g}$ , $s_{g}^{b}$ }, where the firm selects “high green,” and the government selects the regulatory policy, the government’s perceived gain is $\pi_{g}^{b}+U$ . The firm’s perceived benefit in this case is $\pi_{e}^{g}$ . The perceived benefits of the enterprise and government are $\pi_{e}^{n}$ and $\pi_{g}^{a}$ , respectively. When the strategy is { $s_{e}^{n}$ , $s_{g}^{a}$ }, the corporation selects “low green,” and the government selects the incentive policy. When the strategy is { $s_{e}^{n}$ , $s_{g}^{b}$ }, the government selects the regulatory policy and the perceived benefit is $\pi_{g}^{b}$ , whereas when the enterprise selects “low green,” the perceived benefit is $\pi_{e}^{n}-P^{\prime }$ . Table 3 lists the prospective payment matrix for each decision scenario.

**Table 3:** Game payment matrix for the government and enterprise.

	High green (*g*)		Low green (*n*)	
	Enterprise (*x*)	Government (*y*)	Enterprise (*x*)	Government (*y*)
Incentive policies (*a*)	$\pi_{e}^{g}+G^{\prime }$	$\pi_{g}^{a}+U-G_{g}$	$\pi_{e}^{g}$	$\pi_{g}^{a}$
Regulatory policy (*b*)	$\pi_{e}^{n}$	$\pi_{g}^{b}+U$	$\pi_{e}^{n}-P^{\prime }$	$\pi_{g}^{b}$

## 4. Model analysis

### 4.1. Replicated dynamic equation

The game analysis was performed by replicating the dynamics based on the prospect payment matrix calculated based on the prospect theory and the assumptions above. When the enterprise implements the “high green” strategy, $s_{e}^{g}$ , the prospect gain is *u_g_*; when the enterprise implements the “low green” strategy, $s_{e}^{n}$ , the prospect gain is *u_n_*:


(11) $$u_{g}=q\left(\pi_{e}^{g}+G^{\prime }\right)+\left(1-q\right)\pi_{e}^{g}$$

and


(12) $$u_{n}=q\pi_{e}^{n}+\left(1-q\right)\left(\pi_{e}^{n},G^{\prime }\right)$$

The average prospective gain for enterprise is $\bar{u_{e}}$ :


(13) $$\bar{u_{e}}=pu_{g}+\left(1-p\right)u_{n}$$

As a result, the firm’s replicated dynamic equation can be calculated as follows:


(14) $$\begin{array}{l} F\left(p\right)=\frac{dp}{dt}\\ \;\;=p\left(1-p\right)\left(u_{g}-\bar{u_{e}}\right)\\ \;\;=p\left(1-p\right)\left(u_{g}-u_{n}\right)\\ \;\;=p\left(1-p\right)\left(\begin{array}{l} \left(q\left(\pi_{e}^{g}+G^{\prime }\right)+\left(1-q\right)\pi_{e}^{g}\right)\\ -(q\pi_{e}^{n}+\left(1-q\right)\left(\pi_{e}^{n}-P^{\prime }\right) \end{array}\right)\\ \;\;=p\left(1-p\right)\left(\left(G^{\prime }-P^{\prime }\right)q+\pi_{e}^{g}-\pi_{e}^{n}+P^{\prime }\right) \end{array}$$

The prospective gain is *u_a_* when the government implements an incentive policy and *u_b_* when the government implements a regulatory policy:


(15) $$u_{a}=p\left(\pi_{g}^{a}+U-G\right)+\left(1-p\right)\pi_{g}^{a}$$

and


(16) $$u_{b}=p\left(\pi_{g}^{b}+U\right)+\left(1-p\right)\pi_{g}^{b}$$

The average prospective gain for the government is $\bar{u_{g}}$ :


(17) $$\bar{u_{g}}=qu_{g}+\left(1-q\right)u_{n}$$

Based on this, the replication dynamics equation for the government can be obtained as follows:


(18) $$\begin{array}{l} G\left(q\right)=\frac{dq}{dt}\\ \;=q\left(1-q\right)\left(u_{a}-{\bar{u}}_{g}\right)\\ \;=q\left(1-q\right)\left(u_{a}-u_{b}\right)\\ \;=q\left(q-1\right)\left(\begin{array}{l} p\left(\pi_{g}^{a}+U-G_{g}\right)+\left(1-p\right)\pi_{g}^{a}\\ -\left(p\left(\pi_{g}^{b}+U\right)+\left(1-p\right)\pi_{g}^{b}\right) \end{array}\right)\\ \;=q\left(1-q\right)\left(G_{g}p+\pi_{g}^{b}-\pi_{g}^{a}\right) \end{array}$$

The two replicated dynamic Eqs 14, 18 were combined to obtain a 2-D dynamic system of firm and government decisions as follows:


(19) $$\left\{ \begin{array}{c} F\left(p\right)=\frac{dp}{dt}=p\left(1-p\right)\left(\left(G^{\prime }-P^{\prime }\right)q+\pi_{e}^{g}-\pi_{e}^{n}+P^{\prime }\right)\\ G\left(q\right)=\frac{dq}{dt}=q\left(1-q\right)\left(G_{g}p+\pi_{g}^{b}-\pi_{g}^{a}\right)\; \end{array}\right.$$

### 4.2. Analysis of evolutionary stability strategy

According to the aforementioned model, the proportion of businesses adopting a “high green” approach is partially stable when *p* = 0, *p* = 1, or $q=\frac{\pi_{e}^{n}-\pi_{e}^{g}-P^{\prime }}{G^{\prime }-P^{\prime }}\left(G^{\prime }\neq P^{\prime }\right)$ ; the proportion of governments selecting an incentive policy is similarly partially stable when *q* = 0, *q* = 1, or $p=\frac{\pi_{g}^{a}-\pi_{g}^{b}}{G_{g}}$ .When $q=\frac{\pi_{e}^{n}-\pi_{e}^{g}-P^{\prime }}{G^{\prime }-P^{\prime }}\left(G^{\prime }\neq P^{\prime }\right)$ , *p* takes any value, which is the evolutionary stability strategy (ESS) of the system; if $q\neq \frac{\pi_{e}^{n}-\pi_{e}^{g}-P^{\prime }}{G^{\prime }-P^{\prime }}$ or $G^{\prime }=P^{\prime }$ , *p* = 0, and *p* = 1 are the two ESSs of the system; when $G^{\prime }\neq P^{\prime }$ , $P^{\prime }<G^{\prime }$ and $G^{\prime }-P^{\prime }\leq \pi_{e}^{n}-\pi_{e}^{g}$ , then *p* = 1 is the ESS of the system; and if $G^{\prime }\neq P^{\prime }$ , $P^{\prime }>G^{\prime }$ and $G^{\prime }-P^{\prime }\geq \pi_{e}^{n}-\pi_{e}^{g}$ , then *p* = 1 is the ESS of the system.

When $p=\frac{\pi_{g}^{a}-\pi_{g}^{b}}{G_{g}}$ , *q* is the system’s ESS regardless of its value; if $p\neq \frac{\pi_{g}^{a}-\pi_{g}^{b}}{G_{g}}$ , *q* = 0 and *q* = 1 are the system’s two ESSs; and when $\pi_{g}^{a}-\pi_{g}^{b}\leq G_{g}$ , *q* = 1 is the system’s ESS.

The 2-D decision dynamics system of the firm and the government, as reported above, has five local stability points, which are (1,1), (0,0), (1,0), (0,1) and $\left(\frac{\pi_{e}^{n}-\pi_{e}^{g}-P^{\prime }}{G^{\prime }-P^{\prime }},\frac{\pi_{g}^{a}-\pi_{g}^{b}}{G_{g}}\right)$ .

**Figure 2:**
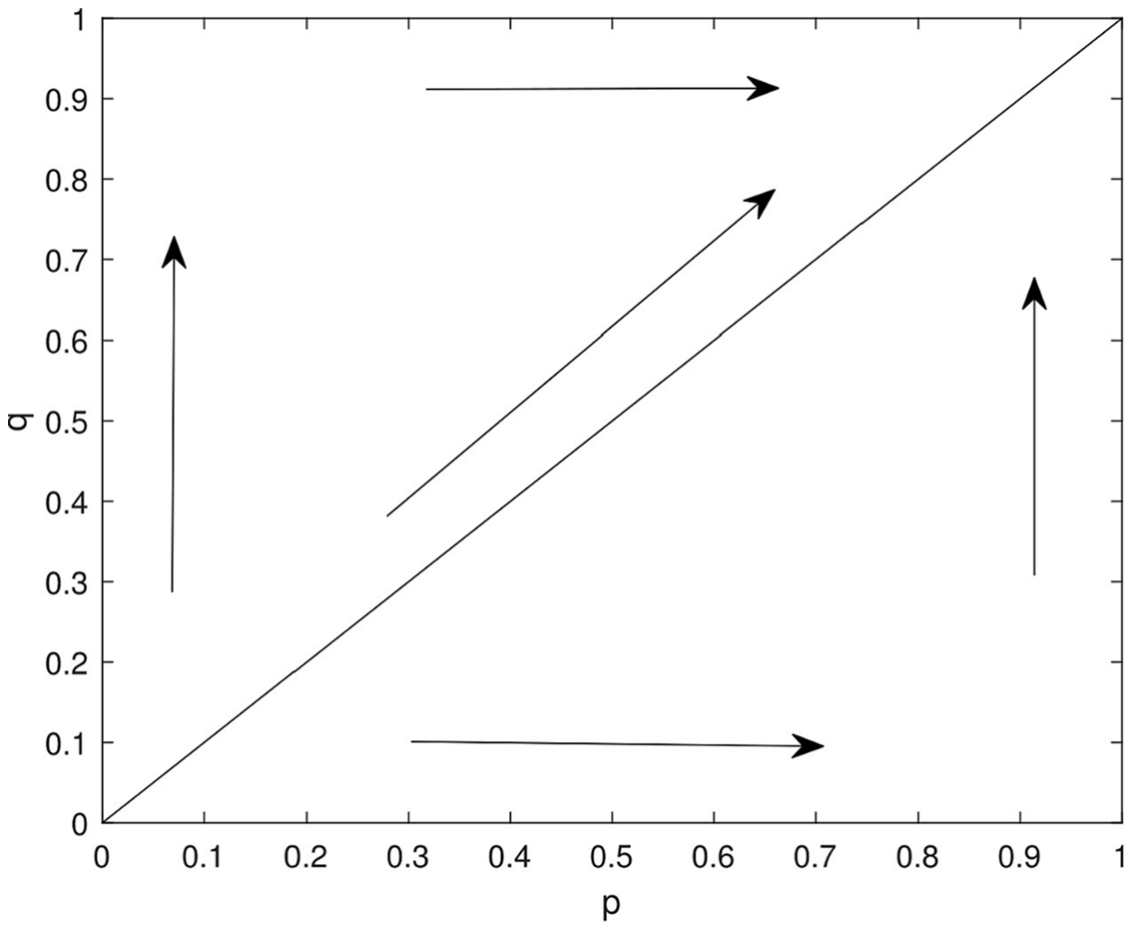
Dynamic evolution phase diagram of the enterprise and government game.

Figure 2 reveals four local stability points in the two-dimensional dynamical system involving the corporation and government. Each equilibrium point corresponds to an evolutionary game strategy that holds practical significance.

The point (1,1) indicates that all enterprises in the system adopt a “high green” strategy, and the government provides incentives to each enterprise. This scenario leads to positive development for both the enterprise and the government, facilitating the achievement of green goals.

The point (0,0) signifies that all enterprises in the system adopt “high green” strategies, and the government takes punitive measures against each enterprise. As a result, all enterprises adopt the “high green” strategy, which causes a decrease in their revenue. However, the government gains revenue from the firm’s green strategy, leading to an increase in the firm’s risk.

The point (1,0) implies that all enterprises in the system adopt a “low green” strategy, while the government continues to provide incentives to each firm. In this case, the government incurs higher costs, and the enterprises receive subsidies but are unable to achieve their green goals.

The point (0,1) indicates that all enterprises in the system adopt a “low green” strategy and the government takes punitive measures against each firm. In this case, there is no additional risk for both the government and enterprise, but no further greening is achieved. These findings shed light on the practical implications of the different equilibrium points in the system, highlighting the dynamics between the corporation and government in pursuing environmentally friendly strategies.

## 5. Simulation

### 5.1. Simulation analysis data

In summary, several factors affect the stability of this evolutionary game system. From the replicated dynamic equation system, $\pi_{e}^{g}$ , $\pi_{e}^{n}$ , $\pi_{g}^{a}$ , $\pi_{g}^{b}$ , *G*, *P*, and other factors can affect this evolutionary game system; furthermore, *p_g_*, *p_n_*, *q_a_*, and *q_b_* influence $\pi_{e}^{g}$ , $\pi_{e}^{n}$ , $\pi_{g}^{a}$ , $\pi_{g}^{b}$ , and other factors. We used MATLAB to simulate the components, examined the development of business and governmental strategy under various parameter modifications, and better understood the impact of each aspect on the evolutionary game system. Considering current circumstances, the initial values of each parameter were established in Table 4.

**Table 4:** Benefits and costs for businesses and governments.

Enterprise benefits		Enterprise costs		Government benefits		Government costs	
*R*_1_	25.2425	*C*_1_	22.2001	*G*_1_	28.8945	*K*_1_	27.6055
*R*_2_	29.7946	*C*_2_	21.0868	*G*_2_	31.0399	*K*_2_	27.9461

We assumed that the “high green” firm had benefit *R*_1_ and cost *C*_1_; the “low green” firm had benefit *R*_2_ and cost *C*_2_; the incentive policy had benefit *G*_1_ and cost *K*_1_; and the regulatory policy had benefit *G*_2_ and cost *K*_2_, where the expected probability of occurrence of each prospect was *p_g_ =* 0.65, *p_n_* = 0.7, *q_a_* = 0.70, and *q_b_* = 0.88.

We conducted calculations and produced the prospect values of enterprises and governments under various strategies based on the prospect theory model utilized in the preceding section, which were $\pi_{e}^{g}$ = 0.77, $\pi_{e}^{n}$ = 2.3155, $\pi_{g}^{a}$ = 0.3856, and $\pi_{g}^{b}$ = 1.0675.

According to the survey research, assuming an initial value of 0.4 for *p* and 0.6 for *q*, in the current social context (He et al., 2021), the proportion of “high green” supply chain enterprises was relatively low while the proportion of government incentives for “high green” environmentally friendly enterprises was significant.

### 5.2. Simulation analysis of factors affecting evolution

**Figure 3:**
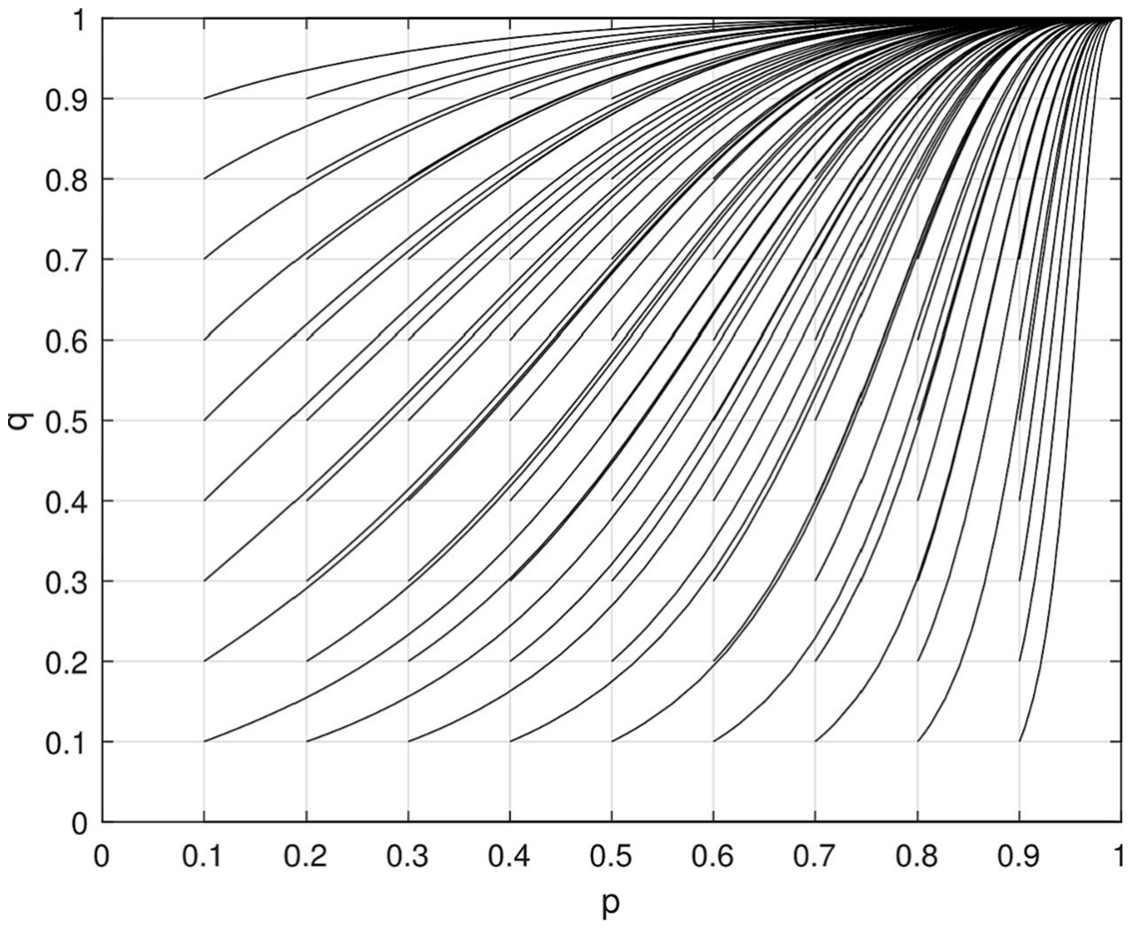
ESS of the enterprise and government evolutionary game simulation.

To simulate the evolutionary game model in MATLAB, we set the value of the government subsidy, *G* = 8, and the value of the government penalty, *p* = 3, for “low green” enterprises. This resulted in Figure 3, which is consistent with the phase diagram change in the equilibrium point analysis mentioned above.

**Figure 4:**
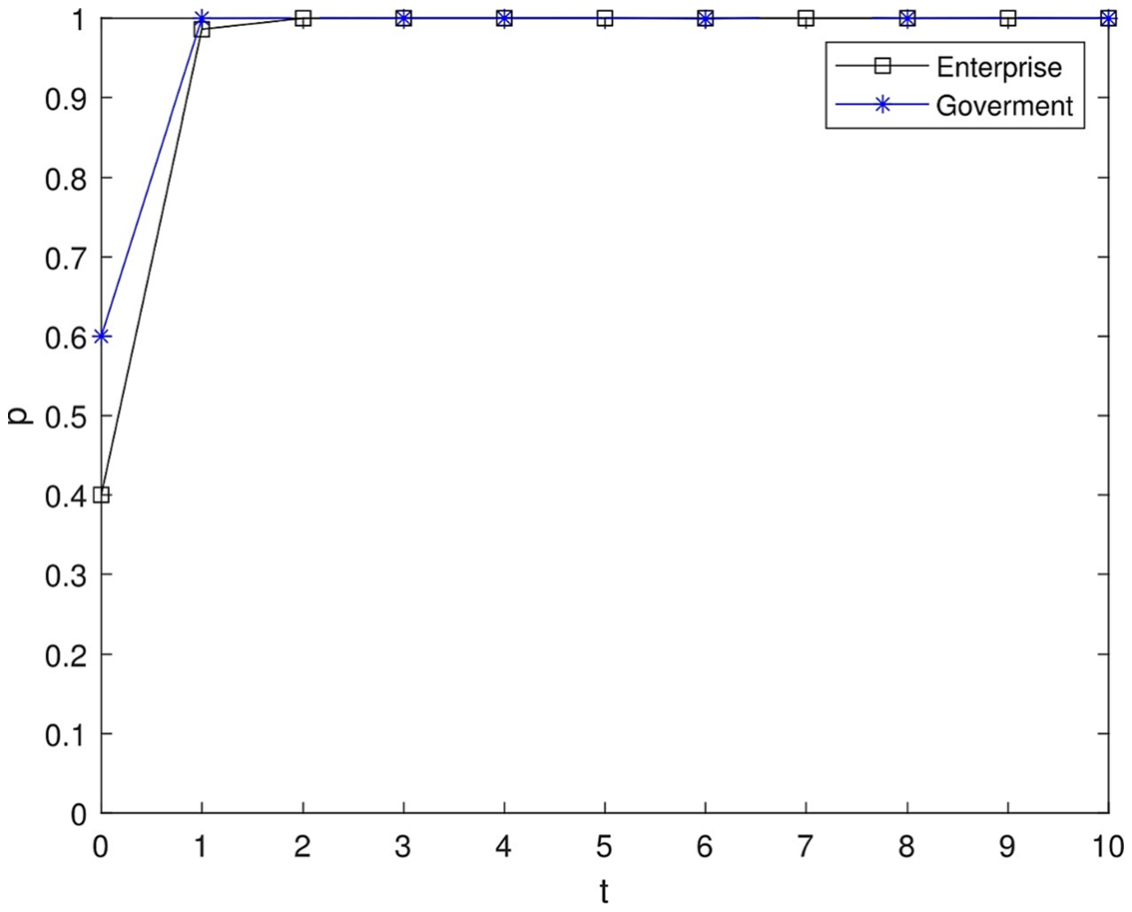
Evolutionary path of government and enterprise strategy.

Figure 4 illustrates the progression of the “high green” strategy as depicted in the simulation. Over time, there was a convergence toward a ratio of 1 between businesses adopting the “high green” strategy and governments implementing incentive policies. Similarly, the ratio of businesses choosing the “high green” strategy and the ratio of governments opting for incentive policies also converged to 1. Remarkably, the government’s incentive policy (reward) continued to rise until it reached a ratio of 1 before the businesses’ choice of the “high green” approach. This clearly demonstrates the significant role of the government in directing environmental protection efforts.

**Figure 5:**
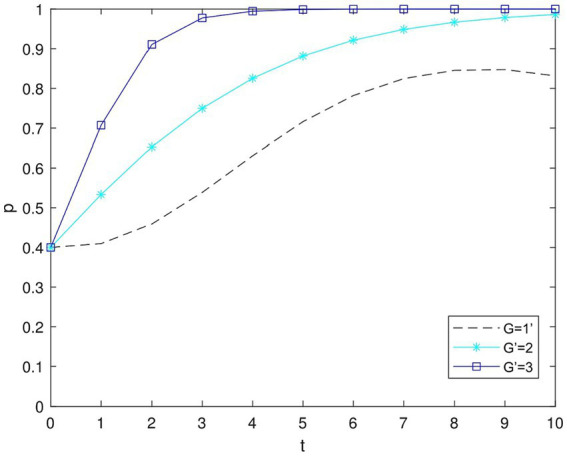
Impact of *G*′ on enterprise decision making.

**Figure 6:**
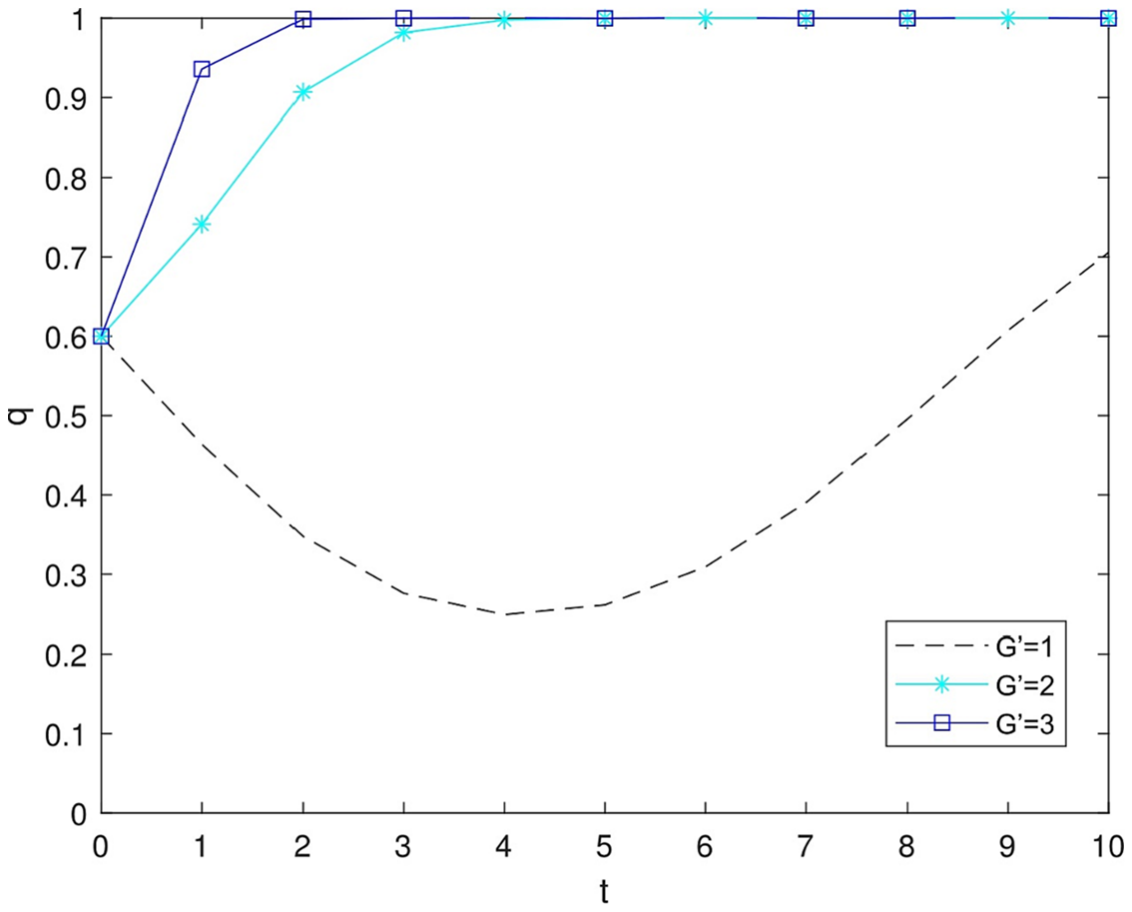
Impact of *G*′ on government decision making.

As depicted in Figures 5, 6, when the perceived value *G*′ of government subsidies increases, enterprises begin to develop in the direction of high environmental sustainability, and the government becomes more inclined to provide subsidies to them. In other words, as the perceived prospect of subsidies grows, enterprises are more willing to adopt the “high green” strategy, leading to a stable game system. This outcome arises from the fact that the enterprises’ perception of increased government subsidies motivates them to engage more actively in environmental protection activities.

**Figure 7:**
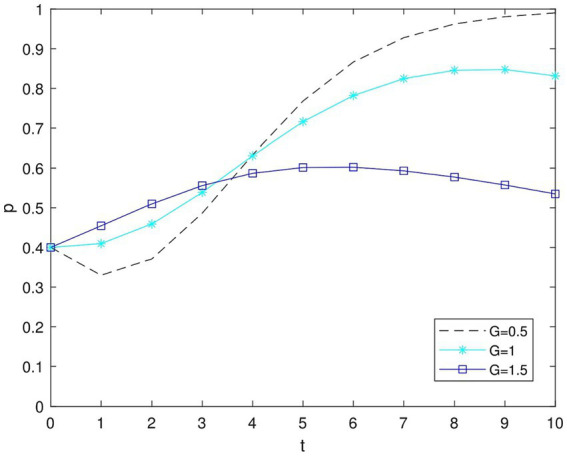
Impact of perceived value of government subsidies on enterprises’ decisions at *p* = 1.

According to Figure 7, when *p* > 0, the government penalizes low green enterprises, and their behavior initially moves in the direction of becoming more environmentally friendly. However, as the magnitude of *G* (government penalties) increases beyond a certain threshold, these enterprises begin to shift back toward low green decisions. In other words, businesses are more likely to adopt a “high green” approach and the game system achieves stability when the government offers fewer subsidies to businesses. Nevertheless, the percentage of businesses choosing the “high green” strategy starts to decline once the number of subsidies reaches a particular threshold. Simply stated, government incentives can partially encourage businesses to engage in green practices, but excessive incentives might lead businesses to become overly reliant on them, resulting in reduced environmental investment or even the use of environmentally destructive methods to boost profits. For instance, the Canadian government decided to give Germany’s Volkswagen a $13 billion subsidy. Governments’ green product incentives could backfire (He et al., 2021), when formulating policies; the government must carefully consider the amount and timing of subsidies to prevent negative impacts on enterprise environmental behavior.

**Figure 8:**
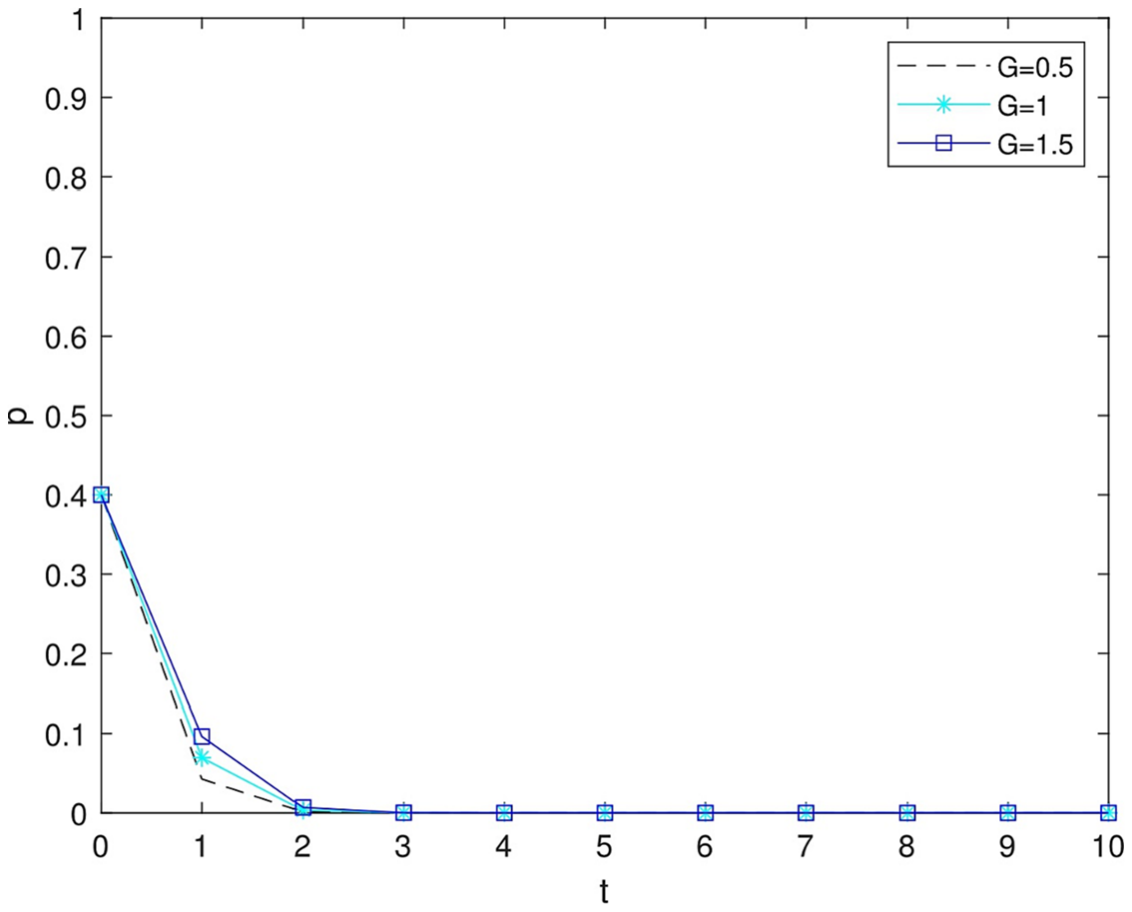
Impact of perceived value of government subsidies on enterprises’ decisions at *p* = −1.

Figure 8 illustrates that when *p* ≤ 0 the government decides not to fine the enterprises, or, in cases where there are no penalties, enterprises are less inclined to pursue high green supply chain plans. The time required for the system to reach a steady state increases as the actual subsidy amount, *G*, from the government to the firm rises. Clearly, government penalties for businesses can effectively encourage them to opt for green supply chains; sanctions against businesses with inadequate environmental standards can lead to the more successful adoption of greener practices compared to simple subsidies.

## 6. Discussion

### 6.1. Main findings

This study obtained three main findings. First, attaining an optimal balance between corporate conduct and government policies proves to be a considerable challenge within the actual functioning of supply chain businesses. Businesses often tend to produce only in line with the minimum green criteria. Four ideal limitations that can lead to the supply chain enterprise evolving to its best form are revealed by the mathematical modeling analysis: when *P*′ is less than *G*′ and *G*′ minus *P*′ is greater than $\pi_{e}^{n}$ minus $\pi_{e}^{g}$ ; when *P*′ is greater than *G*′ and *G*′ minus *P*′ is less than the difference of $\pi_{e}^{n}$ minus $\pi_{e}^{g}$ , *p* is equal to the value of $\pi_{g}^{a}$ minus $\pi_{g}^{b}$ divided by *G_g_*; and when *P*′ is not equal to *G*′, and *q* is equal to $\pi_{e}^{n}$ minus $\pi_{e}^{g}$ minus *P*′ divided by *G*′ minus *P*′. However, in reality, the business environment of supply chain enterprises and the strategic environment of the government are highly complicated. The government’s reward and punishment mechanism are not yet perfect, and decision makers in enterprises may exhibit various behavioral biases because of overconfidence or cognitive biases. Considering the reflection effect in prospect theory, it becomes challenging for all these factors to align simultaneously. Moreover, relying solely on government subsidies proves insufficient to achieve the best outcomes.

Second, our research identified the main factors influencing the government’s decision to provide subsidies to green supply chain businesses. Due to the complex and uncertain nature of the game system between green supply chain enterprises and the government, we identified six key influencing factors that affect the effectiveness of subsidies. These factors were examined from the perspectives of both enterprises and the government: $\pi_{e}^{g}$ , $\pi_{e}^{n}$ , $\pi_{g}^{a}$ , $\pi_{g}^{b}$ , *G* and *P,* respectively. The simulation revealed that increasing the prospective value of the “high green” strategy, reducing the prospective value of the “low green” strategy, boosting government subsidies, and imposing higher penalties for “low green” enterprises can indeed partially encourage the adoption of the “high green” strategy. However, it is crucial to recognize that there are specific thresholds for the impact of *G* and *P* on the adoption of environmental protection strategies. Only by making rational decisions based on these criteria can we effectively accelerate and promote the transformation of green supply chain enterprises.

Last but not least, both governmental fines and moderate subsidies have demonstrated effectiveness in promoting the adoption of green supply chains. However, the provision of substantial government subsidies falls short in incentivizing businesses to opt for sustainable supply chains. The model’s results demonstrate that the penalty system is a valuable tool for the government to encourage businesses to opt for green supply chains. A win-win situation for the government and enterprises can be attained through modest government subsidies. Conversely, excessive subsidies may not only fail to achieve the desired effect of promoting enterprises’ green supply chains but also lead to losses for both the enterprises and government. In other words, government green subsidies to businesses should be capped and primarily utilized to penalize environmentally damaging businesses.

### 6.2. Theoretical implications

This study notably enhances the existing literature on public support for sustainable supply chains through several significant contributions. First, our study broadens the theoretical understanding of green supply chains by introducing the perspective of psychological and behavioral factors. Previous studies mainly focused on the cost-effectiveness of green supply chains and variables influencing businesses’ environmental behavior, such as competitive pricing and green supply chain strategies, but largely missed behavioral decision-making (Knemeyer and Naylor, 2011; Barman et al., 2021). In this context, we transfer the research focus to psychological and behavioral factors, and investigate how these factors impact the dynamics within a green supply chain. We then use an innovative approach that combines the prospect theory with evolutionary game theory to construct an evolutionary game model for both businesses and governments involved in a green supply chain. Existing research on green supply chain decision models typically considers elements such as profit, cost, benefit, and government subsidies for energy-efficient products (Xue et al., 2019), but often overlooks the influence of firm behavior on subsidy results. To fill this gap, we integrate prospect theory and evolutionary game theory to provide a more comprehensive analysis involving both government and firm behavior. This combined perspective paves the way for new insights and methodologies for devising green supply chain policies. A key assumption underpinning our study is that every decision-maker operates with finite rationality, exhibits loss aversion, and is sensitive to psychological effects. Moreover, it is expected that these decision-makers will choose the most advantageous course of action, based on the perceived value of their prospects.

Second, our research substantially extends the prevailing theoretical work on green supply chains, offering fresh insights into government subsidy mechanism design. By utilizing prospect theory in conjunction with an evolutionary game model, we pushed forward the field of evolutionary game research concerning government subsidies for green supply chains. Historically, studies have primarily concentrated on how government subsidies affect businesses or stressed aspects of collaboration, negotiation, and equity issues within green supply chains (Adhikari and Bisi, 2020; Ye et al., 2022). To address these real-world concerns more effectively, our study harmonizes prospect theory and evolutionary game theory from the synergistic perspectives of both businesses and governments. We crafted an evolutionary game model encapsulating the dynamics between green supply chain enterprises and governments. This model scrutinizes how businesses negotiate decisions regarding government subsidies, along with assessing the sway of government incentives and regulations over the adoption of green practices. In scenarios where government subsidies are not factored in, enterprises may lean towards a “high green” strategy only when they perceive the prospects of this choice outweighing a “low green” approach. However, firm decisions are subject to influence by government conduct when considering its impact on supply chain enterprises. The model we have developed demonstrates that government policies significantly shape businesses’ propensity to adopt “high green” strategies. Government subsidies can inspire businesses to select “high green” methods, and escalating fines for “low green” firms under regulatory policies can stimulate the embrace of greener production models.

Finally, we delve deeper into the intricacies of the connection between green supply chain decisions and governmental subsidies. Our research contributes to the formulation of effective government subsidy strategies by aligning them with varied strengths of subsidies and associated punitive measures. The central theme focuses on how governments can more effectively incentivize businesses to adopt eco-friendly, green supply chains. Building upon previous research, it is evident that government subsidies pave the way for businesses to embrace green production techniques, concurrently reducing consumer costs (Meng et al., 2021a). In our study, we scrutinize the potential future benefits for both businesses and governments using the lens of prospect theory, leveraging these insights to develop an evolutionary game model tailored for green supply chain enterprises and governments. However, our findings also indicate a paradox; large government subsidies often fail to motivate businesses toward green supply chains, resulting in losses for both parties. Therefore, this paper delivers theoretical frameworks for governments to stimulate the transformation of green supply chain businesses into sustainable, environmentally conscious organizations, thus aiding their transition. In summary, our research provides crucial insights into behavioral decision-making within subsidized green supply chains, while underscoring the significance of incorporating prospect theory and evolutionary game theory when assessing the efficacy of government subsidies.

### 6.3. Practical implications

#### 6.3.1. Providing appropriate subsidy

According to the status of supply chain enterprises, the government should provide appropriate subsidies. Offering small government subsidies can encourage businesses to adopt green supply chains, leading to a significant increase in both government and businesses’ earnings. This benefits not only the growth of businesses but also advances both social and economic development. For enterprises that meet the requirements, the government may extend financial assistance in the form of tax breaks, preferential loan terms, and capital subsidies. These efforts aim to enhance their competitiveness and foster growth momentum. Additionally, the government should focus on developing more detailed environmental policy measures, enabling businesses to execute them more effectively. This will create a conducive environment for supply chain enterprises to thrive safely and orderly. Simultaneously, the government should actively expand its support and oversight of businesses to foster sustainable development. This approach will enable supply chain businesses to address challenges effectively and achieve long-term growth.

#### 6.3.2. Penalizing non-environmental enterprises

By imposing fines on enterprises with low or no environmental standards, the government should encourage corporations to take their environmental obligations more seriously. Enforcing sanctions on businesses that do not prioritize environmental protection or lack eco-friendly practices will also compel such enterprises to pay greater attention to environmental issues. For instance, the government can employ administrative sanctions, such as fines and the cancellation of enterprise licenses, to penalize businesses that disobey environmental laws. These measures will hold businesses accountable for their transgressions and foster a heightened awareness of the importance of environmental preservation. This approach creates a positive feedback loop between the economy, environment, and society, while simultaneously promoting the sustainable development of businesses.

#### 6.3.3. Enhancing communication and environmental awareness

Prospect theory suggests that the perception of business revenue under environmentally friendly techniques is influenced by enterprise culture, as decision-makers often exhibit non-fully rational characteristics. To achieve sustainable development, the enterprise should increase their environmental consciousness, actively invest in environmental protection, and adopt eco-friendly techniques. Moreover, businesses should be mindful of and positively respond to the government’s environmental regulations to achieve better win-win outcomes.

To promote environmental protection, the government and businesses must improve communication and exchange ideas, engaging in collaborative debates on environmental policies. Cooperation and coordination should serve as the foundation for supporting the growth of the environmental protection industry. Additionally, the government must enhance its oversight to ensure that businesses comply with environmental protection laws, thus contributing to the healthy development of the environmental protection sector.

In conclusion, government subsidies play a significant role in encouraging enterprises to transition to sustainable supply chains. However, such subsidies must be carefully implemented, considering factors including the environment, economics, and policy implications. Proper regulation and evaluation are essential for their effectiveness.

### 6.4. Limitations and future research

There are several limitations to this study. First, this study lacks a discussion on how internal green supply chain enterprise procedures influence enterprise decision-making. Factors such as enterprise culture and staff understanding have a significant impact on how businesses manage their green supply chains. Hence, it is imperative to incorporate these elements into the research scope and develop a more comprehensive model of green supply chain management (Han et al., 2022; Huo et al., 2023). Second, the discussion of government subsidies in this paper is relatively brief and fails to delve into the specific forms, criteria, and implementation methods of these subsidies. To comprehend the effects of government subsidies on green supply chain businesses and suggest more sustainable incentive programs, additional empirical research is required. Finally, this study overlooks critical aspects of model-building, such as regional disparities and social network connections between specific businesses, which may limit the model’s applicability. Therefore, it is essential to consider more factors in the model-building process and expand the research horizon to enhance the applicability and prediction accuracy of the model.

To address the aforementioned issues and gain a deeper understanding of green supply chains, a future study can be conducted on the mechanisms of cooperation between businesses and governments in the context of green supply chains as per the current situation. This investigation should further explore the form, level, substance, and process of cooperation between businesses and the government to develop a more comprehensive model of green supply chain management. Moreover, the sustainability of government subsidies must be considered to ensure their long-term impact on green supply chain businesses. To develop a more realistic subsidy model, it is essential to analyze the actual effects of various forms, standards, and implementation techniques on businesses.

Furthermore, the effects of government subsidies on different types of enterprises, sizes, geographic regions, and other factors should be studied to determine more effective subsidy policies. When building a green supply chain management model, several factors, including the internal mechanisms of enterprises, should be considered. For example, social network relationships, organizational structure, management mode within the enterprise, geographical factors, and the policy environment can all contribute to improving the applicability and prediction accuracy of the model. To comprehensively analyze the effectiveness and feasibility of green supply chain management models, a variety of methods and techniques, including statistical analysis, case studies, and simulations, should be employed. This approach will enable a more robust assessment of these models. By addressing these issues and adopting a comprehensive research approach, we can enhance our understanding of green supply chains and contribute to the development of sustainable practices in the supply chain industry.

## 7. Conclusion

This study utilized prospect theory and evolutionary games to examine the impact of government subsidies on the behavior of green supply chain businesses and the government. We have considered behavioral and psychological factors and examined whether its potential an impact on decision-making behavior between governments and businesses in green supply chain practices. We have obtained the following results based on the analysis.

First, we observed that achieving an optimal balance between corporate conduct and government policies proves to be a considerable challenge within the practical operation of supply chain businesses, which is consistent with the finding of Stekelorum et al. (2022). This finding implies that the government’s reward and penalty mechanisms are not yet well-developed, and corporate decision-makers might exhibit various behavioral deviations owing to overconfidence or cognitive biases. Consequently, businesses might frequently align their production only with the minimum green criteria.

Second, our research identified that the perceived benefits of enterprises, the magnitude of government subsidies to enterprises, and the penalties imposed by government to enterprises, are the primary factors influencing the government’s decision to grant subsidies to green supply chain businesses. These factors have a huge impact on how complex and uncertain the game between green supply chain enterprises and the government is. Therefore, the Chinese government can develop relevant policies to guide and provide proper incentives to improve enterprises’ green supply chain practices.

Third, both governmental fines and moderate subsidies have been emerged as having a positive correlation with promoting the adoption of green supply chains. Interestingly, the provision of substantial government subsidies falls short of incentivizing businesses to opt for sustainable supply chains. The model’s results demonstrate that the penalty system is a valuable tool for the government to encourage businesses to opt for green supply chains. This finding indicates that a win-win situation for government and enterprises can be attained through modest government subsidies. Conversely, excessive subsidies may not only fail to have the intended impact of boosting green supply chains but also have losses for both the enterprises and the government.

In conclusion, the research results indicate that government behavior can not only affect whether enterprises adopt green supply chains, but also effectively enhance the benefits for both the government and the enterprises. Thus, we propose incentive policies that better align with the actual circumstances faced by businesses, thus holding significant practical application potential. The incentive programs presented here offer practical applications that are better suited to the real-world challenges encountered by businesses.

## Data availability statement

The original contributions presented in the study are included in the article/supplementary material, further inquiries can be directed to the corresponding author.

## Author contributions

LH: Supervision, Conceptualization, Methodology, Writing – original draft. YZ: Methodology, Writing – original draft. CW: Supervision, Writing – review & editing. JS: Supervision, Writing – review & editing.
